# Near infra-red labelling and tracking of corneal endothelial cells in-vivo

**DOI:** 10.1038/s41598-022-09677-w

**Published:** 2022-04-15

**Authors:** Maninder Bhogal, Heng-Pei Ang, Shu-Jun Lin, Chan N. Lwin, Khadijah Adnan, Gary Peh, Jodhbir S. Mehta

**Affiliations:** 1 Tissue Engineering and Cell Therapy, Singapore Eye Research Institute, Singapore, Singapore; 2 Cornea Unit, Guy’s & St Thomas’, London, UK; 3 Eye-ACP, Duke-NUS Graduate Medical School, Singapore, Singapore; 4 Singapore National Eye Centre, Singapore, Singapore; 5 School of Material Science and Engineering, Nanyang Technological University, Singapore, Singapore

**Keywords:** Preclinical research, Translational research

## Abstract

Following corneal transplantation, there is an initial, rapid decline in corneal endothelial cells (CECs) following surgery. Direct imaging of post-transplantation endothelial cells is only possible weeks after surgery and with a limited field of view. We have developed a labelling approach using 1,1′-dioctadecyl-3,3,3′,3′-tetramethylindotricarbocyanine iodide (DIR) dye solution, that enables tracking of labelled CECs in vivo for at least 1 month. Initial in vitro optimization, with assessments of dye concentration on fluorescence, cellular toxicity and cell migration, performed in propagated primary CECs. Subsequently, in vivo evaluation of cellular labelling was assessed within a rabbit wound healing model. Finally, real-time visualization of human cadaver donor tissue incubated in DIR transplanted into rabbits was achieved using a clinical confocal microscope. Results revealed detectable fluorescence increased with concentration to a plateau of 100 µg/ml, with no toxicity of CECs at any concentration evaluated. DIR-labelled CECs were detectable in vivo up to 1 month, and transplanted labelled donor graft could be visualized and were trackable in vivo. Acute endothelial rejection in 1 rabbit was evidenced by detectable DIR positive cells within the anterior chamber. DIR imaging allowed for detailed imaging of the transplanted human corneal endothelium, and enabled non-invasive observation of the corneal endothelial morphology following transplantation.

## Introduction

After corneal transplantation, a rapid decline in corneal endothelial cell density (ECD) is observed in the early post-operative period^1,2^. We have previously shown that iatrogenic cell loss occurs during the preparation^3^, storage^4^, insertion and unfolding of endothelial grafts^5^. In addition, factors such as air tamponade^6,7^, post-operative inflammation and co-morbidities such as glaucoma^8,9^, are likely to affect early endothelial cell loss. Immediately after transplantation, areas of dead or denuded endothelial cells are present^5^. A process of cell migration and restoration of the endothelial monolayer must be completed in order for corneal clarity and thickness to be restored^10^.

Monitoring of endothelial cells can either be performed directly, by imaging the cells^10^, or indirectly, by using measurements of endothelial function: central corneal thickness (CCT) and transparency^11^. Presently, endothelial cells are imaged in vivo in the clinical setting using specular or confocal microscopy^12^, although newer modalities, based on optical coherence tomography (OCT) imaging, are being developed^13^. Typically, only a small number of cells, between 30 to 100, are imaged at high magnification^14^. Image quality can be poor following endothelial keratoplasty (EK), especially with non-contact methods^15^. Poor image acquisition from off-axis portions of the cornea, and the lack of automated methods for stitching multiple images together, mean these methods do not allow for global assessment of the entire graft^16^. Consequently, tracking small areas of interest over time can be very difficult^17^. In both specular and confocal microscopy, image quality is reliant on a clear corneal stroma, meaning images obtained through oedematous or scarred corneas are often of poor quality. The presence of an intra-cameral gas bubble can also impact image quality. As such, it is often not possible to use these methods in the first 2 weeks following EK, nor is it possible to use these methods to examine endothelial healing following wounding studies, which result in significant swelling of the cornea^5^.

Wounding studies are commonly used to assess the efficacy of surgical/pharmaceutical interventions aimed at promoting endothelial healing^15–17^. In our previous studies, in vivo endothelial wound healing was assessed using repeated intracameral trypan blue injection^10^. Whilst this was a reliable and reproducible technique, it is an invasive procedure that carries the risk of damaging healing endothelial cells or introducing infections^18^. The method cannot be used in the early post-operative periods following EK or Descemet’s membrane transplantation, whilst an intracameral gas bubble remains. Caution needs to be taken early after bubble resolution to avoid dislodging the recently transplanted material. An additional limitation of the trypan blue technique is that it only allowed global imaging of endothelial wound healing. This meant animals had to be sacrificed at staggered time intervals in order to perform histological evaluation of wound healing at a cellular level.

We previously developed and validated a method to image endothelial cells, in vivo, using Calcein AM; a green-fluorescent viability stain^5^. Whilst capable of global and cell-level imaging in the immediate post-operative period, our in vitro experiments showed that Calcein AM was rapidly cleared from endothelial cells indicating that any useful fluorescence signal will be extinguished by 24 h post-incubation. As such, it is not useful for longer-term tracking of endothelial cells in vivo. The fate of the transplanted cells can only truly be determined if methods to quantify transplanted corneal endothelial cell death immediately post-operatively can be combined with techniques to track endothelial cells through early post-operative phase, which we define as 1 month based on previous reports^19,20^.

In this study, we describe a technique for labelling and tracking corneal endothelial cells in vivo, both globally across the entire cornea and at an individual cell level. We compared these in vivo images to immuno-histology following animal sacrifice. We show this technique is suitable for tracking both native and transplanted endothelial cells and can be used in wound healing studies as well as xenotransplantation of human EK grafts into rabbits.

## Results

### Optimizing DIR concentration in vitro

**Figure 1:**
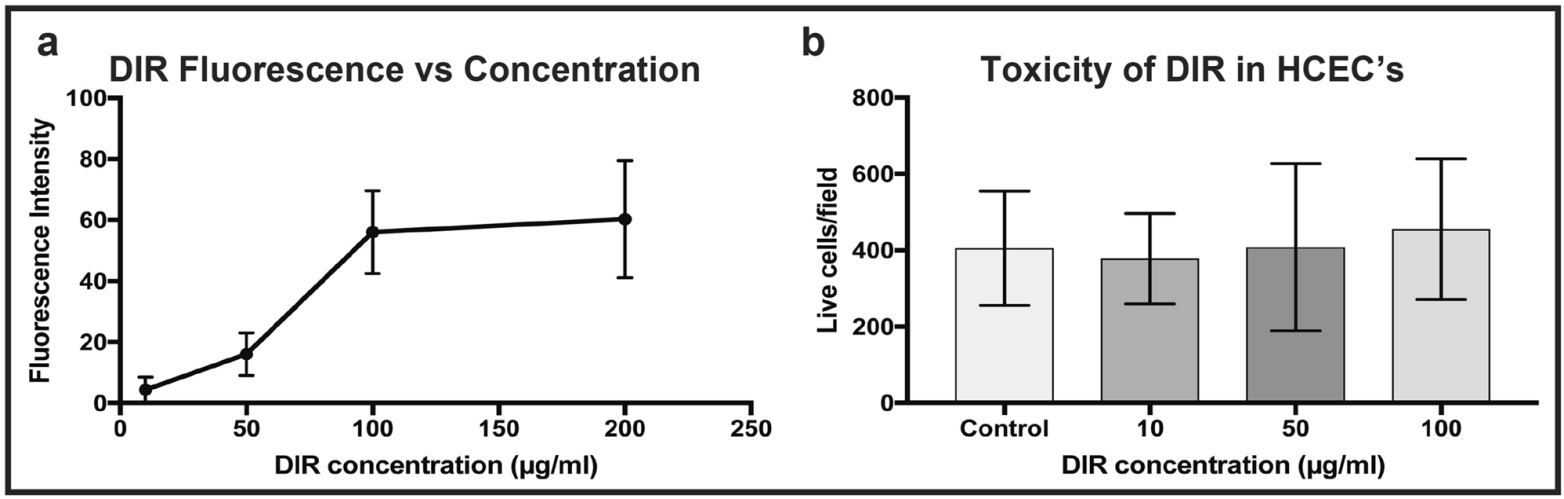
Optimization of DIR concentration in vitro. (**a**) Graph showing average (mean and standard deviation) DIR fluorescence in human CECs as a function of concentration. (**b**) Bar graph comparing toxicity of DIR at different concentrations in cells derived from the same donor cornea (n = 6) per concentration.

**Figure 2:**
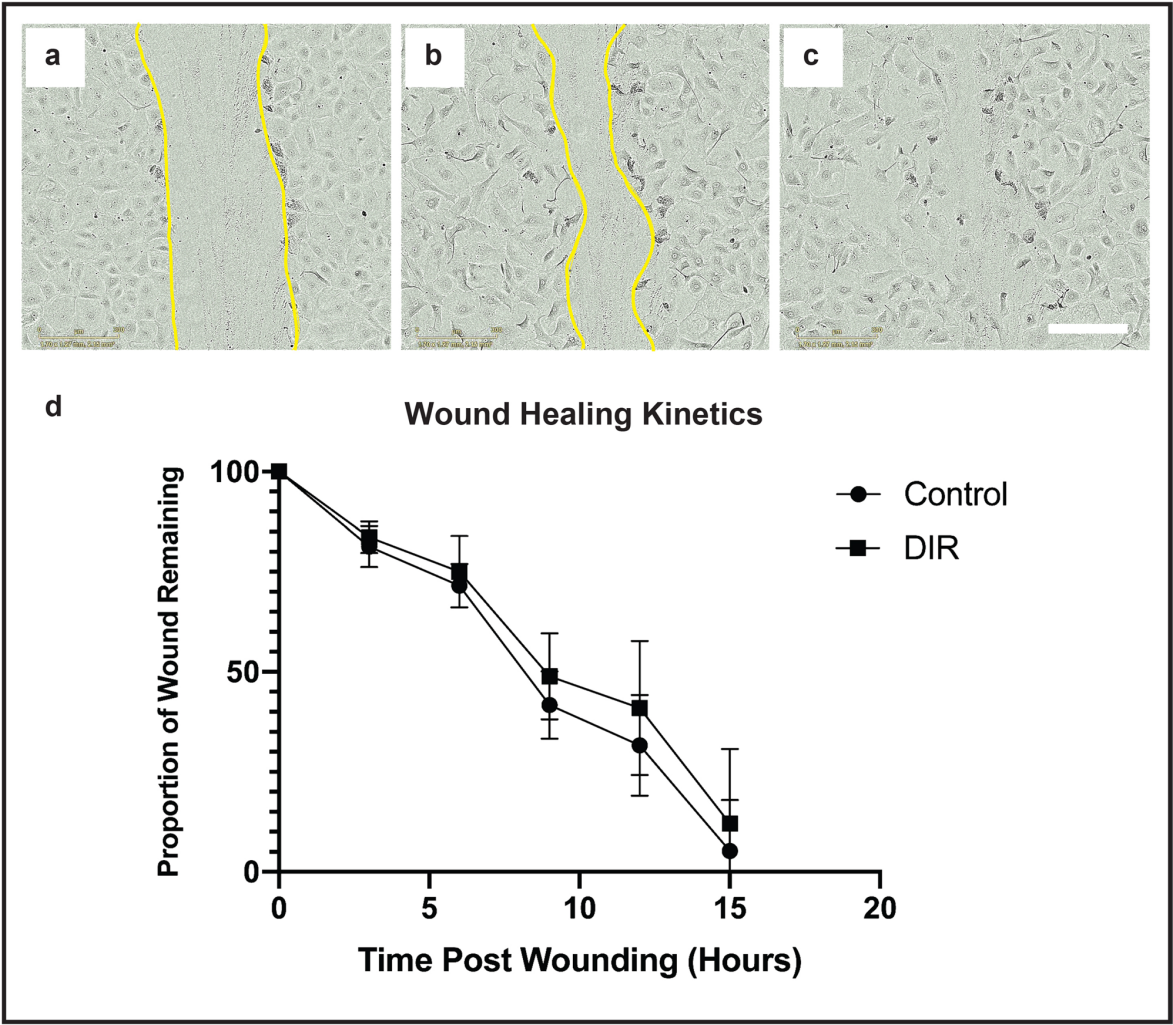
Wound healing in DIR-labelled primary human CECs. (**a**) Human CECs were plated and allowed to stabilize. A standard scratch wound was made with a 10 µl pipette tip. Th original wound area was delineated (yellow border) and the wound area measured. Time-lapse images were taken every hour after wounding. (**b**) The wound area was reassessed serially (yellow border), and the percentage wound area remaining calculated. (**c**) Imaging was continued until complete closure of the scratch wound was observed. (**d**) The wound area remaining (mean and standard deviation) was compared between paired CEC controls vs CEC exposed to DIR every 3 h for 15 h. (N = 6, paired t-test). No significant difference between controls and DIR stained cells was seen at any time point. Scale bar: 250 µm.

Average DIR fluorescence increased with concentration in a linear-quadratic fashion. Comparison of average fluorescence levels at different doses, derived from macroscopic images taken from individual wells, revealed a significant difference between cells stained with 50 µg/ml and 100 µg/ml (repeated measures ANOVA, corrected p < 0.0001, N = 15 per group) but not between 100 and 200 µg/ml (p = 0.78) (Fig. 1a). Based on these findings a dose of 100 µg/ml was chosen for in vitro subsequent labelling. No significant differences were found on Calcein AM/ethidium homodimer toxicity assessment between DIR at any concentration and matched controls from the same donor (N = 6) (Fig. 1b). Wound healing kinetics following standardized scratch wounding in HCEC’s were similar between control and treated groups (Fig. 2). There was no significant difference in wound area remaining at any time point (n = 6, paired t-test) (Fig. 2d).

### In vivo labelling of rabbit endothelial cells

**Figure 3:**
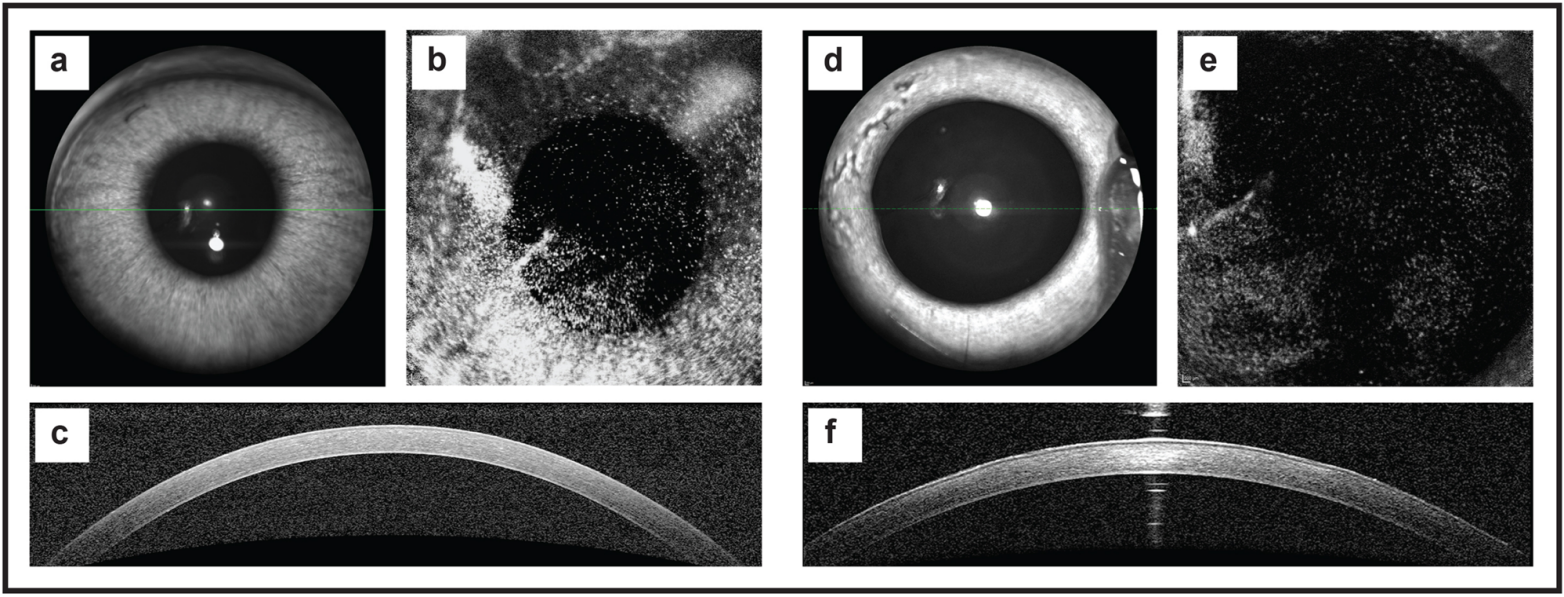
The effect of DIR on rabbit corneal endothelium following intracameral injection in vivo. DIR (final concentration 100 µg/ml) was injected into the anterior chamber of 3 rabbits to determine if this had any side-effects. (**a**) Photomicrograph of the rabbit anterior segment taken 1 day post DIR intracameral injection. The cornea remains transparent, with easy visualization of the iris details. (**b**) Infra-imaging using, the ICG filters on the Spectralis HRA, shows variable fluorescence across the endothelium, with speckled hyperfluorescent seen at areas of increased cellular dye uptake. (**c**) OCT image showing an optical cross-section of the central rabbit cornea. No increase in corneal thickness or inflammation is seen (CCT. Images from the same rabbit cornea taken on day 28 post DIR injection. (**d**) A photomicrograph of the rabbit anterior segment showing the cornea remains clear. (**e**) Infra-imaging using the ICG filters shows residual fluorescence at day 28, although maximal fluorescence intensity is reduced. (**f**) An OCT image taken at day 28 showing corneal thickness remains normal and unchanged throughout the study period (CCT 377 µm).

Direct injection of DIR diluted in the rabbit aqueous to a final concentration of 100 µm/ml was successful in labelling rabbit endothelial cells. No adverse effects were observed following DIR injection, with no significant inflammation (conjunctival hyperemia, iris vascular dilation, anterior chamber cellular activity) seen at any time point. Slit lamp microscopy and infra-red imaging on day 1 showed no change in corneal clarity (Fig. 3a). DIR stained endothelial cells were visible on infra-red imaging, with significant cell-to-cell variation in staining intensity observed (Fig. 3b). There was no increase in corneal thickness (Fig. 3c,f) at any time point after surgery, with CCT remaining < 400 µm throughout the observation period. At day 28, all corneas remained clear (Fig. 3d). DIR labelled cells were still visible, but with some reduction in observable fluorescence (Fig. 3e). DIR uptake was prominent in the iris, meaning that pupil dilation was necessary to reduce background fluorescence in subsequent examinations. Background fluorescence was further reduced by imaging using the higher magnification lenses, as these reduced the depth of focus (Fig. 3e).

### In vivo corneal wounding: comparison between in vivo DIR imaging, confocal microscopy and immunohistology

**Figure 4:**
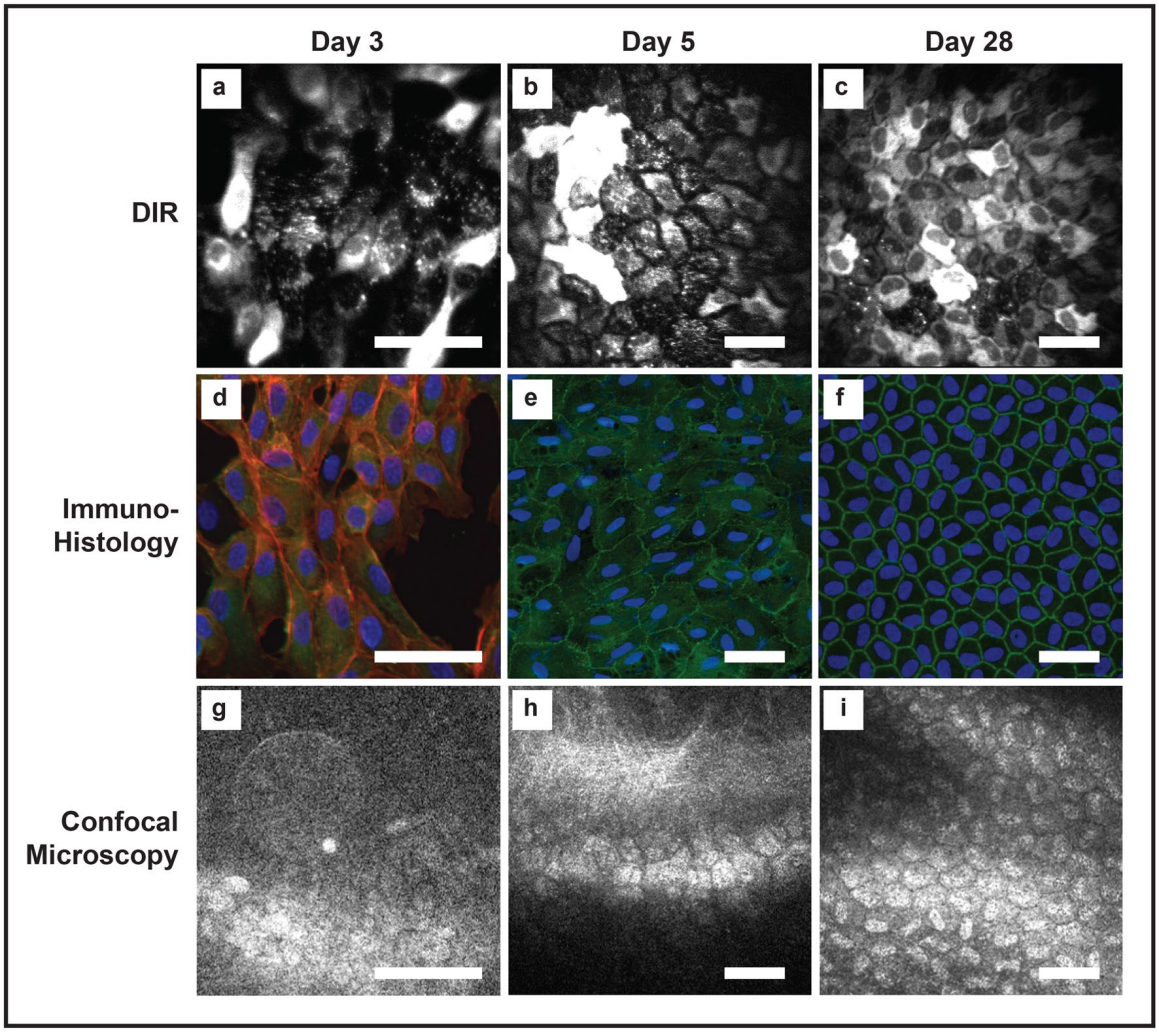
In vivo tracking of corneal endothelial cells. (**a**–**c**) The top row shows DIR stained rabbit corneal endothelial cells at day 3, 5 and 28 post wounding, imaged in vivo. (**d**–**f**) The middle row shows the same cells imaged with immunohistology at the same time points, after the animal was sacrificed. (**g**–**i**) The bottom row shows images acquired with conventional in vivo confocal microscopy. Each column represents images taken from the same cornea at the same time point (**a**) In vivo DIR imaging at day 3 showing migrating endothelial cells at the leading edge of the wound. Cells show an elongated morphology with incomplete monolayer formation. Marked variation in DIR uptake is seen between adjacent endothelial cells, with some staining intensely (**b**) At day 5, a complete monolayer is seen with high degrees of pleomorphism and broad intercellular junctions. (**c**) By day 28 cells have regained a more regular morphology and tight junctions have become narrower. Fixed cells stained with phalloidin, ZO-1 and Hoechst show similar morphological features to in vivo DIR imaging. (**d**) At day 3 cells are elongated with no junctional ZO-1 staining. (**e**) At day 5 a complete monolayer is seen with incomplete junctional ZO-1 staining and pleomorphic cells. (**f**) At day 28, regular cells with mature borders are seen. (**g**) Conventional in vivo confocal imaging failed to acquire useful images at day 3, and (**h**) day 5. (**i**) At day 28 a stable monolayer of cells was visible on conventional confocal microscopy, but nuclear morphology was more prominent than cell border morphology. Scale bar: 100 µm.

DIR staining was compared to conventional confocal microscopy and post-sacrifice immunohistology in 6 rabbits (2 sacrificed at day 3, 2 at day 5 and 2 at day 28 post wounding). By using additional magnifying lenses attached to the HRA/Spectralis, it was possible to image individual DIR stained endothelial cells, although significant variability in the fluorescence of adjacent endothelial cells was observed (Fig. 4). During early wound healing (day 3), elongated, migrating endothelial cells were seen (Fig. 4a). This same morphology was observed on immunofluorescence staining performed after sacrifice of the same animal (Fig. 4d). At this stage, no endothelial imaging was possible with conventional confocal microscopy (Fig. 4g). There was no difference in cell circularity measured from DIR and immunofluorescence images, with an average value of 0.51 ± 0.14 for histology derived circularity and 0.58 ± 0.12 for DIR imaging (t-test, *p* = 0.34). After wound closure, a confluent monolayer of cells with broad cell borders was seen with in vivo DIR imaging (Fig. 4b). This corresponded to confluent cells with incomplete tight junction formation, as assessed by ZO-1 immunofluorescence staining (Fig. 4e). Conventional confocal imaging was still unable to produce useful images, largely because applanation of the cornea resulted in undulations of the posterior corneal surface (Fig. 4h). After initial wound closure, continuing maturation of central endothelial cells was observed. As time progressed, cell borders became narrower and cells became more circular (Fig. 4c). The regular endothelial mosaic observed with DIR staining at day 28 corresponded to small, largely hexagonal cells, with a continuous ring of ZO-1 staining seen on immunofluorescence (Fig. 4f). Conventional in-vivo confocal images could be obtained from day 14 onward as seen at day 28 (Fig. 4i). Analysis at day 28 showed no difference in cell circularity (0.71 vs 0.74, t-test, *p* = 0.87) or the number of hexagonal cells as assessed by histology and DIR imaging. (50.9% (histology) vs 49.1% (HRA/Spectralis), chi-squared, *p* = 0.9).

### In vivo corneal wounding: comparison between in vivo DIR imaging and trypan blue staining

**Figure 5:**
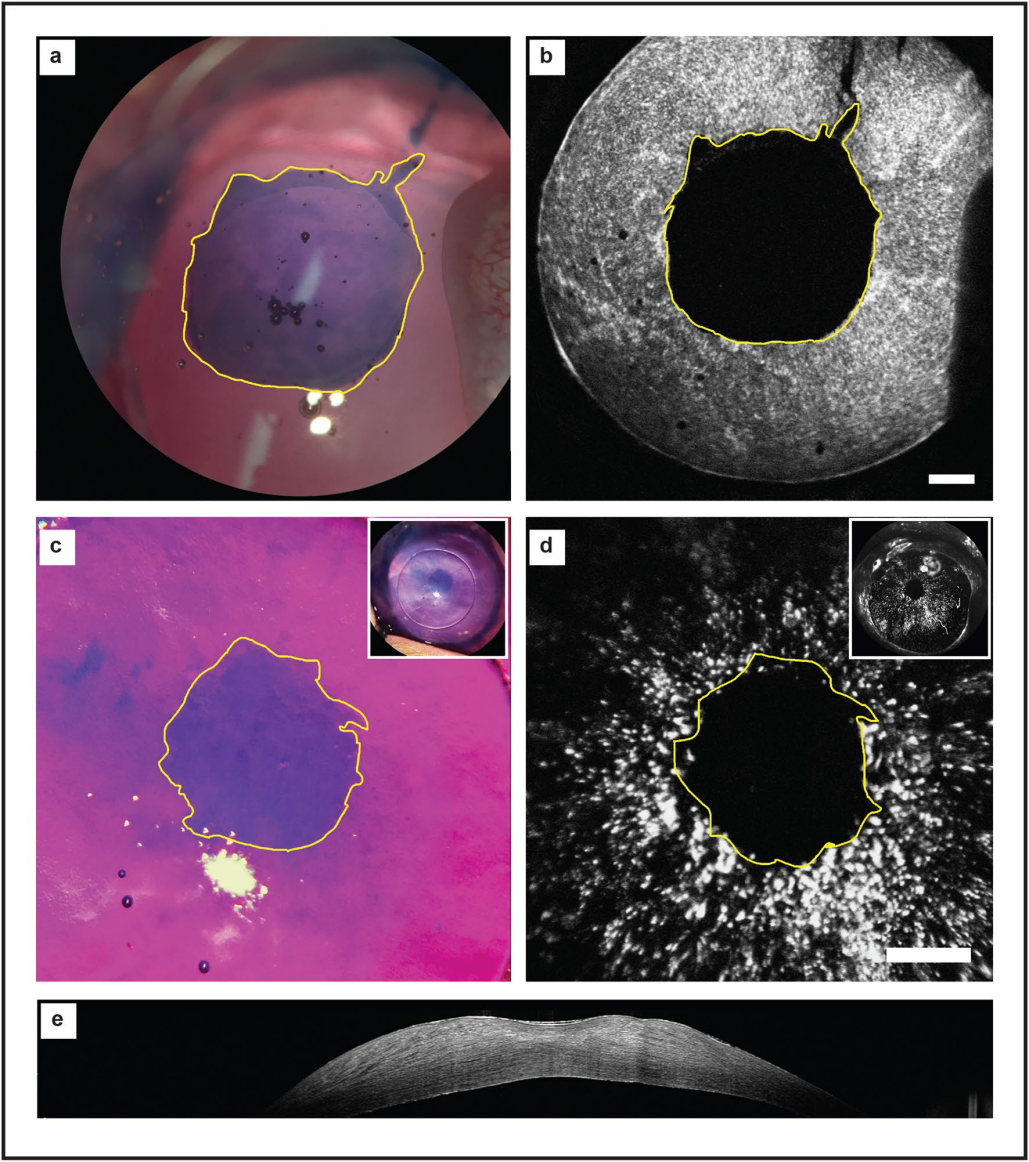
Comparison between in vivo DIR imaging and trypan blue staining. (**a**) Intra-operative trypan blue staining shows bare DM where endothelial cells have been removed by scraping. (**b**) The same eye was imaged immediately post-operatively. The DIR imaging of the area of denuded endothelial cells shows good concordance with intraoperative trypan blue (yellow outline), where DIR fluorescence is blocked by the nictitating membrane. At 3 days post-wounding, significant closure of the wound can be seen. (**c**) Injection of intracameral trypan blue stains a small residual area bare DM (yellow outline). A feathered edge is observed, representing migrating endothelial cells. (**d**) Infrared imaging of the same eye shows the same area of bare DM, surrounded by fluorescent endothelial cells (yellow outline). (**e**) An OCT image of the cornea at day 3 post wounding confirms DIR images are successfully acquired through thickened and irregular corneas. Scale bar: 1 mm.

**Figure 6:**
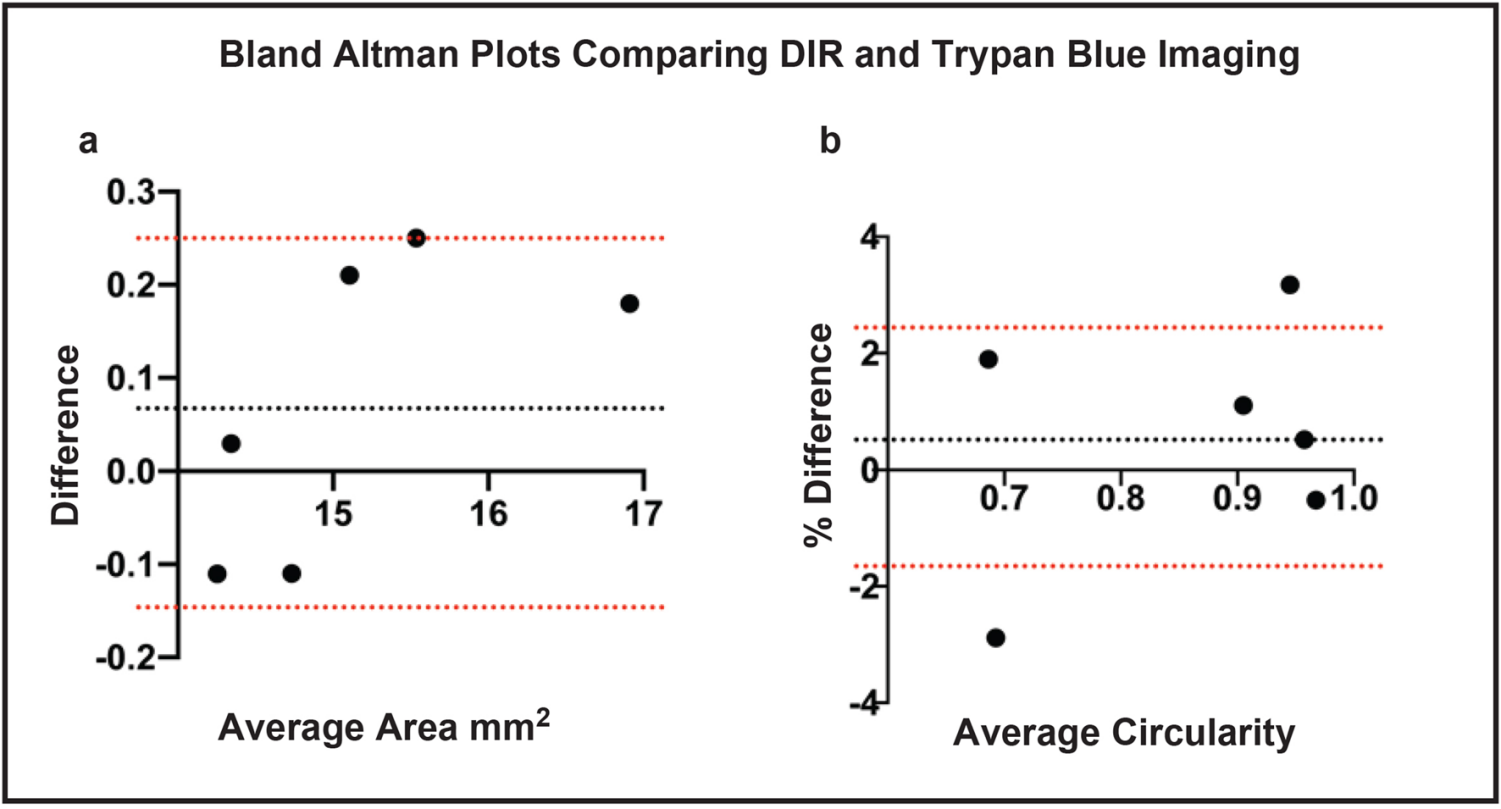
Bland–Altman Plots comparing in vivo wound size as assessed by DIR staining and intra-cameral trypan blue injection. Cornea endothelial wounds were measured using trypan blue and DIR in 6 eyes. The dotted black line represents the average bias, with the wound area measured by trypan blue tending to be marginally larger. The red dotted lines represent the 95% percent confidence interval. (**a**) Residual wound area as measured by DIR and trypan blue was within 0.3 mm for all samples. (**b**) Residual wound circularity as measured by DIR and trypan blue was within 4% for all individual samples.

Good agreement was seen between macroscopic, non-invasive DIR imaging and intracameral trypan blue staining, both at the time of wound creation (Fig. 5a,b) and at day 3 after wounding (Fig. 5c,d). Importantly, it was possible to acquire these images through corneas that were thickened or irregular in shape, as observed on the simultaneously acquired OCT (Fig. 5e). Bland–Altman plots were produced to compare the area and circularity of wounds (Fig. 6). Good agreement was seen between both methods, with less than 2% difference in area seen in any sample (Fig. 6a). Circularity was within 4% for all samples (Fig. 6b).

### In vivo tracking of migrating endothelial cells following endothelial wounding in the rabbit

**Figure 7:**
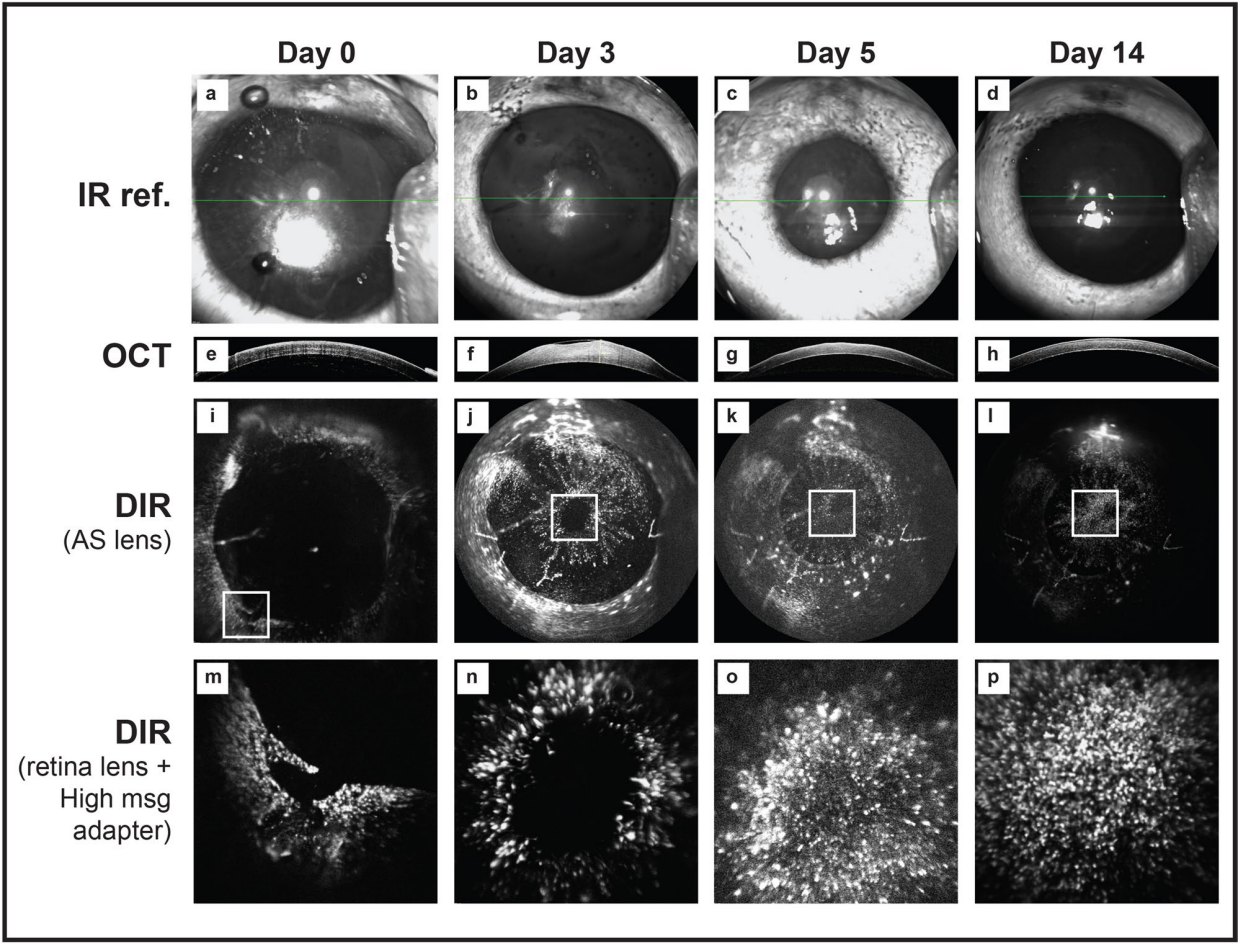
In vivo tracking of endothelial cells at pan-corneal and single-cell level. Images taken from an individual, DIR stained rabbit cornea. Following standard endothelial wounding, infra-red (**a**–**d**), OCT (**e**–**h**) and DIR images (**i**–**p**) were acquired using the HRA/Spectralis. Use of the anterior segment lens supplied by the manufacturer allowed macroscopic imaging of the whole cornea. By coupling additional lenses to the infinity optics retinal lens, higher magnification imaged could be captured (**m**–**p**). The images in the bottom row correspond to the areas seen in the white square outline in the macroscopic images. By sequentially imaging with increasing magnification, it was possible to track areas of interest. Immediately after scraping no DIR positive cells are seen in the wound area (**i**,**m**). At day 3 the cornea is still significant thicker that pre-wounding (**f**). The wound area can be seen to be much smaller (**j**, white box). At higher magnification levels, migrating cells closing the wound at the edge are seen (**n**). By day 5, although some residual corneal oedema and thickening persists on OCT imaging (**g**), complete wound closure can be observed (**k**,**o**). By day 14, corneal thickness has returned to normal (**h**) and a stable layer of DIR positive cells was observed with macroscopic (**i**) and higher magnification imaging (**p**).

The wounding of the rabbit corneal endothelium in vivo was followed for 14 days using infra-red (Fig. 7a–d), OCT (Fig. 7e–h), and DIR imaging (Fig. 7i–l) with their respective magnified region (Fig. 7m–p). Complete closure of an 8 mm endothelial scrape wound occurred by day 5 (Fig. 7k–o). CCT (as assessed by OCT) returned to pre-wound levels by day 14 in all cases (Fig. 7h). These findings are in keeping with our previously published results^10^ obtained from scrape wounding in unstained corneas. Taken together, these findings suggest that DIR staining does not have a deleterious impact on endothelial healing or migration in vivo.

### Labelling of ex vivo human corneas

**Figure 8:**
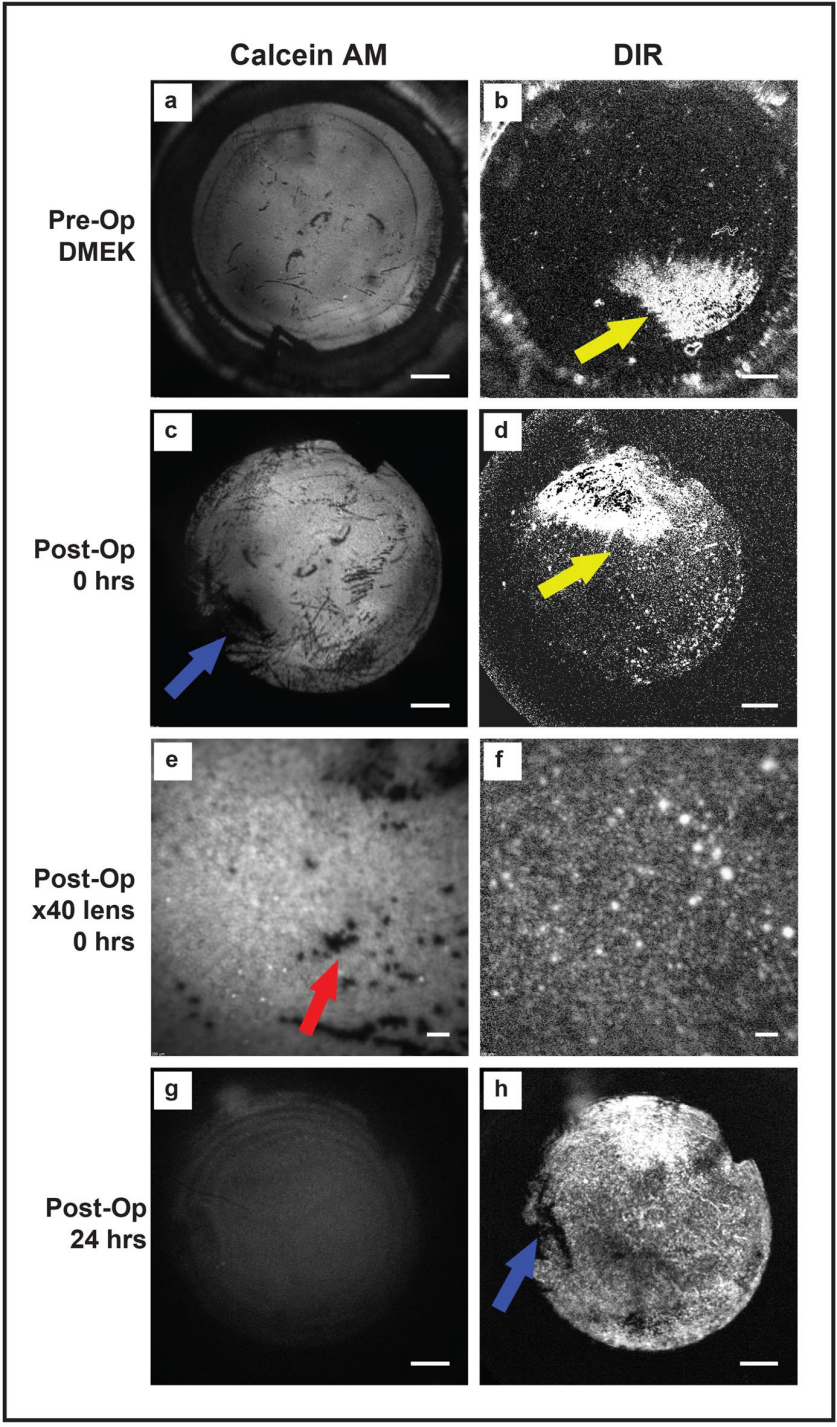
Labelling of ex vivo human corneas. Human corneas were dual stained with Calcein AM and DIR dyes. (**a**) After preparation for implantation, the DMEK graft, resting on stroma, is returned to the viewing chamber and imaged using the FFA filter set to detect Calcein AM stained cells. Note the presence of a triangular orientation mark. Uniform green fluorescence is seen as areas of healthy cells and black areas represent dead or absent cells. (**b**) The same transplant is imaged using the ICG filters to detect DIR staining. There is marked variation in fluorescence across the graft meaning portions become either over or under exposed on global imaging. The yellow arrow points to an area of high DIR fluorescence. Comparison with the Calcein AM staining shows that most of the graft, including this area is cover by viable cells. (**c**) Global Calcien AM imaging of the graft, in vivo, immediately following transplantation shows a new area of cell death attributable to implantation trauma (blue arrow) (**d**) Global DIR imaging of the graft, in vivo, immediately following transplantation shows the same variation of fluorescence (**e**) High magnification imaging acquired by coupling a ×40 air objective microscope lens to the retinal imaging lens of the HFA/Spectralis. Individual Calcein AM negative cells are seen. (**d**) DIR imaging using the same lens shows DIR fluorescence in the cells that appeared non-fluorescent on the global image, suggesting the level of fluorescence is variable, but present across the entire graft. At this time point, comparison between DIR and Calcein AM imaging shows DIR does not differentiate between living and dead, but attached, cells. (**f**) At 24 h post transplantation, Calcein AM fluorescence has diminished to the point where now useful images can be obtained. (**h**) By contrast, at 24 h post-implantation, DIR fluorescence has become more uniform and areas corresponding to pre-identified dead cells can be seen to be devoid of DIR staining, suggesting the cells have been ejected from the monolayer (blue arrow). Scale bar (**a**–**d** and **g**–**h**): 1 mm, Scale bar (**e**,**f**): 250 µm.

Double staining using Calcein AM and DIR allowed multiplexed image acquisition of donor human corneas within the donor viewing chamber (Fig. 8a,b), as well as immediately post operatively inside the rabbit eye (Fig. 8c,d). Initially, staining with DIR showed large degrees of variation, with adjacent areas of the corneal endothelium showing markedly different degrees of fluorescence (Fig. 8b,d, yellow arrows). Calcein AM staining was able to detect large areas of cell loss (Fig. 8c, blue arrow) and differentiate between living and dead individual cells and cells clusters, when a magnifying objective lens was attached to the imaging set-up (Fig. 8e, red arrow). DIR staining was not able to differentiate between living cells and dead endothelial cells that remained attached to DM (DIR +’ve/Calcein am −‘ve) (Fig. 8d,f). As predicted by our in vitro assessment^5^, Calcein AM fluorescence diminished quickly, and no useful fluorescent images could be obtained at 24 h post-implantation (Fig. 8g). Conversely, DIR fluorescence tended to become more uniform by 24 h post graft insertion. At 24 h post implantation, macroscopic areas devoid of cells could be seen, and corresponded to the areas of cell death seen immediately following transplantation using Calein AM (Fig. 8h, blue arrow c.f. Calcein AM Fig. 8c). This suggests that non-vial endothelial cell have been ejected from the transplant by this stage.

### Tracking transplanted human endothelial cells in vivo

**Figure 9:**
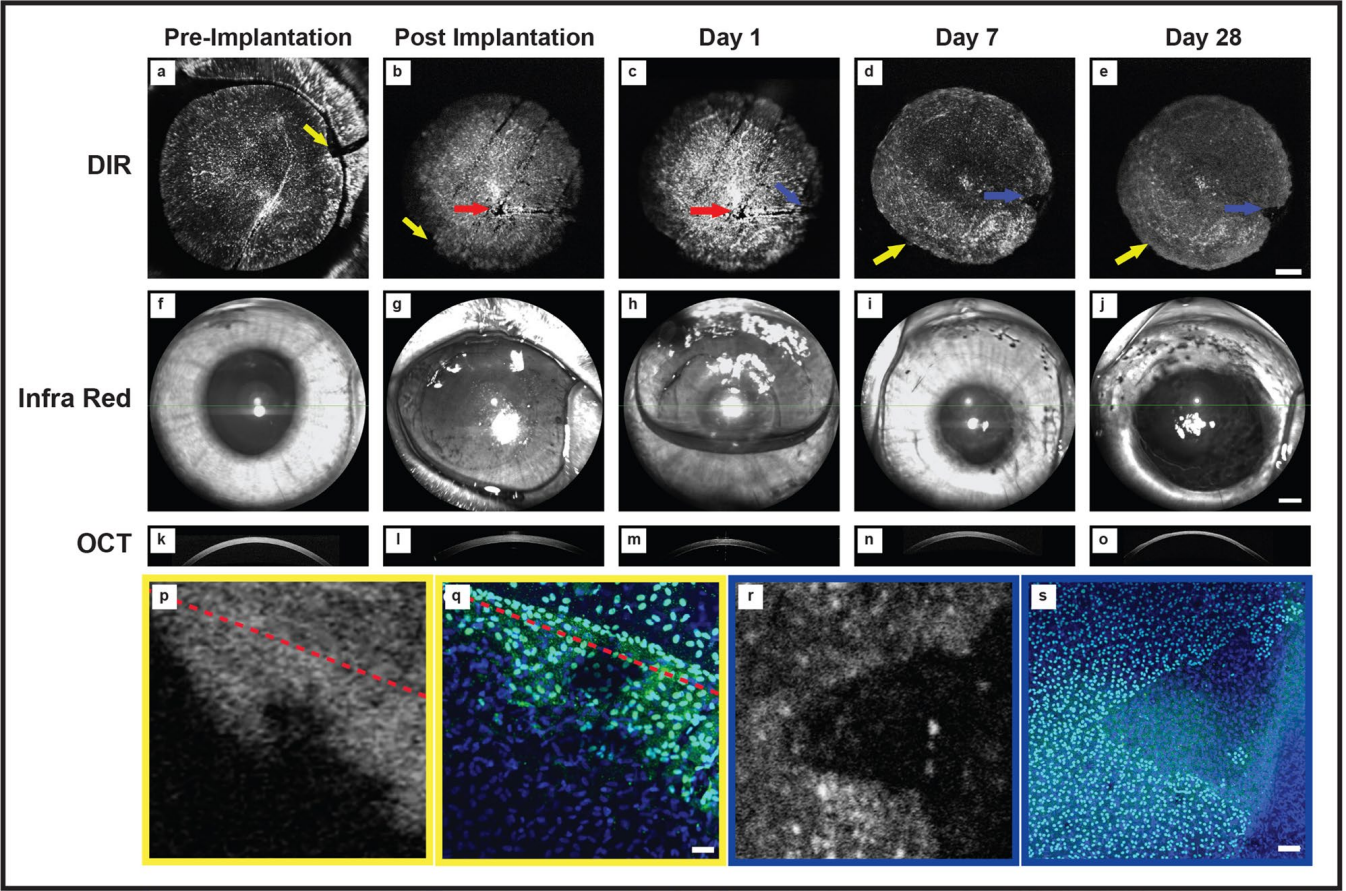
Tracking of transplanted human corneal endothelial cells in vivo. (**a**) A DIR stained DMEK graft imaged in the viewing chamber pre-implantation. A triangular notch is cut out the circular graft and used to confirm the correct side is up during surgery (yellow arrow). (**b**) The same graft is imaged immediately after surgery. Linear, parallel wounds associated with graft insertion can be seen following DMEK (red arrow) as can the notch in the transplanted graft (yellow arrow). (**c**–**e**) The graft is repeatedly imaged over 28 days. Central areas, previously devoid of cells, become DIR positive suggesting cells migration over DM has occurred (red arrow). The triangular notch fades suggesting cells have migrated off the graft and onto the rabbit DM at this point (yellow arrow). Areas in the periphery of the graft are not DIR fluorescent, suggesting they are devoid of human endothelial cells. (**f**–**j**) Images of the rabbit eye show at the time of imaging show that DIR fluorescence is detectable when the anterior chamber contains air (**g**,**h**) and that cornea clarity is restored once a stable endothelial monolayer has been established (**i**,**j**). OCT images of cornea at various time points following transplantation (**k**–**o**). Scale bar (**a**–**j**): 1 mm. (**p**) The triangular notch shows created for orientation identification shows a loss of the straight edge (red dashed line) with apparent migration of endothelial cells off the graft. (**q**) Images of the same area are acquired after staining with human nuclear antibody (FITC). The presence of human endothelial cells beyond the border of the DMEK graft (red dashed line) is confirmed. Scale bar: 250 µm. (**r**) Areas that do not stain with DIR are seen in the graft periphery. (**s**) Cells staining positively with DAPI, but not staining with anti-human nuclear antibody (FITC) can be seen, suggesting these cells are of rabbit origin and that transfer of fluorescence between cells does not occur. Scale bar: 250 µm.

Human EK grafts labelled with DIR were implanted into the rabbit anterior chamber. Stained endothelial cells within the graft maintained strong fluorescence for the entire study period. DIR images of the graft were taken at the pre-implantation (Fig. 9a) and post-implantation (Fig. 9b), and at intervals from day 1 to day 28 (Fig. 9c–e) in conjunction with infra-red (Fig. 9f–j), and OCT (Fig. 9k–o). The yellow bordered figures (Fig. 9p–q) is the magnified area denoted by the yellow arrows (Fig. 9a,b,d,e), whereas the blue bordered figures (Fig. 9r,s) is the magnified area denoted by the blue arrows (Fig. 9c–e). At day 1 post implantation, areas of bare DM devoid of endothelial cells were visible (Fig. 9c; red arrows). A triangular orientation mark (yellow arrow) used to confirm the correct position was also clearly visible. Over the first week, cells migrated to cover the areas of the bare DM (Fig. 9c; red arrows). Areas of cell migration from the donor to host could be seen on DIR imaging. In the case illustrated, this resulted in loss of the straight edge of the triangular orientation mark (yellow arrows). Following animal sacrifice, endothelial cells were stained with anti-human nuclear antibodies (Fig. 9q,s, FITC channel). Migration of human endothelial cells (stained green) across the graft border was visible (Fig. 9q) and the pattern of donor cell distribution showed good concordance between DIR imaging and immunohistological assessment (Fig. 9p,q). Transfer of fluorescence to neighboring rabbit endothelial cells was not observed. Areas of rabbit endothelial migration onto the host DM were seen in DMEK cases (Fig. 9p,r) but not in DSAEK. These cells were distinguishable, by the lack of staining with both DIR (Fig. 9p,r) and anti-human nuclear antibodies (Fig. 9q,s).

**Figure 10:**
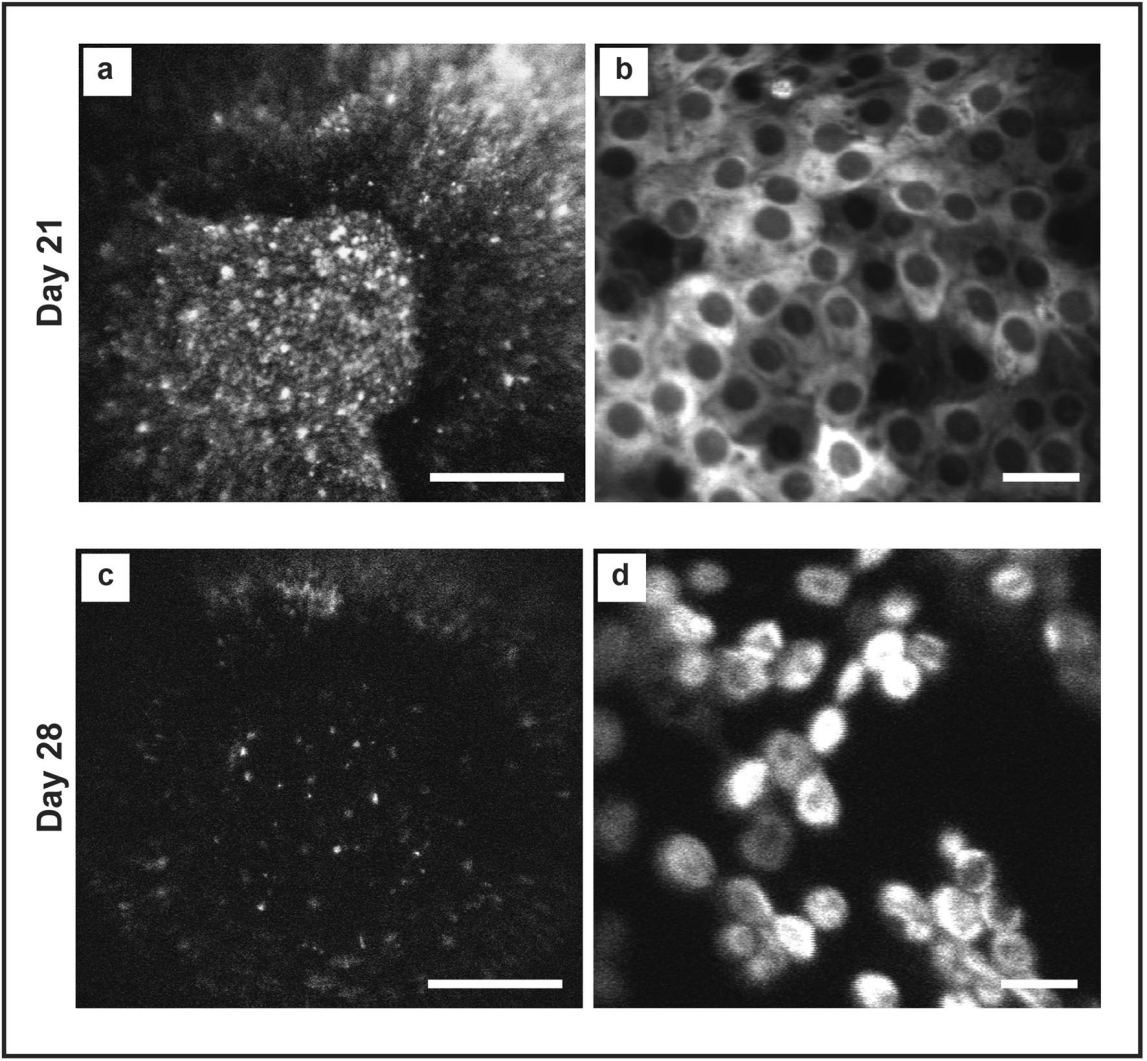
Spectralis images depicting an event of acute graft rejection. (**a**) Low magnification HRA/Spectralis image of a central area of a human DMEK graft, 21 days after transplantation into a rabbit eye. This shows a confluent monolayer of DIR positive cells. Scale bar: 1 mm. (**b**) On day 28 corneal oedema was noted associated with a significant reduction in DIR fluorescence. (**c**) At higher magnifications, a monolayer DIR positive, human endothelial cells were seen at day 21. Scale bar: 50 µm. (**d**) On day 28, the stable monolayer has been replaced with sparse cells that appear rounded and are in the process of detaching from the DM.

In one animal that had DMEK, clinical features of rejection (corneal oedema, ciliary injection) was observed at day 28 (Fig. 10). Compared to day 21 images (Fig. 10a,b), DIR imaging showed central loss of fluorescence at lower magnification (Fig. 10c) and patchy attachment of small, circular endothelial cells at higher powers (Fig. 10d). This was associated with the onset of circulating, DIR positive, cells in the anterior chamber (Video S1). An anterior chamber tap confirmed the presence of cellular material, but the yield was too low to confirm cell origin or DIR positivity using FACS/immunofluorescence.

### High resolution cell imaging

**Figure 11:**
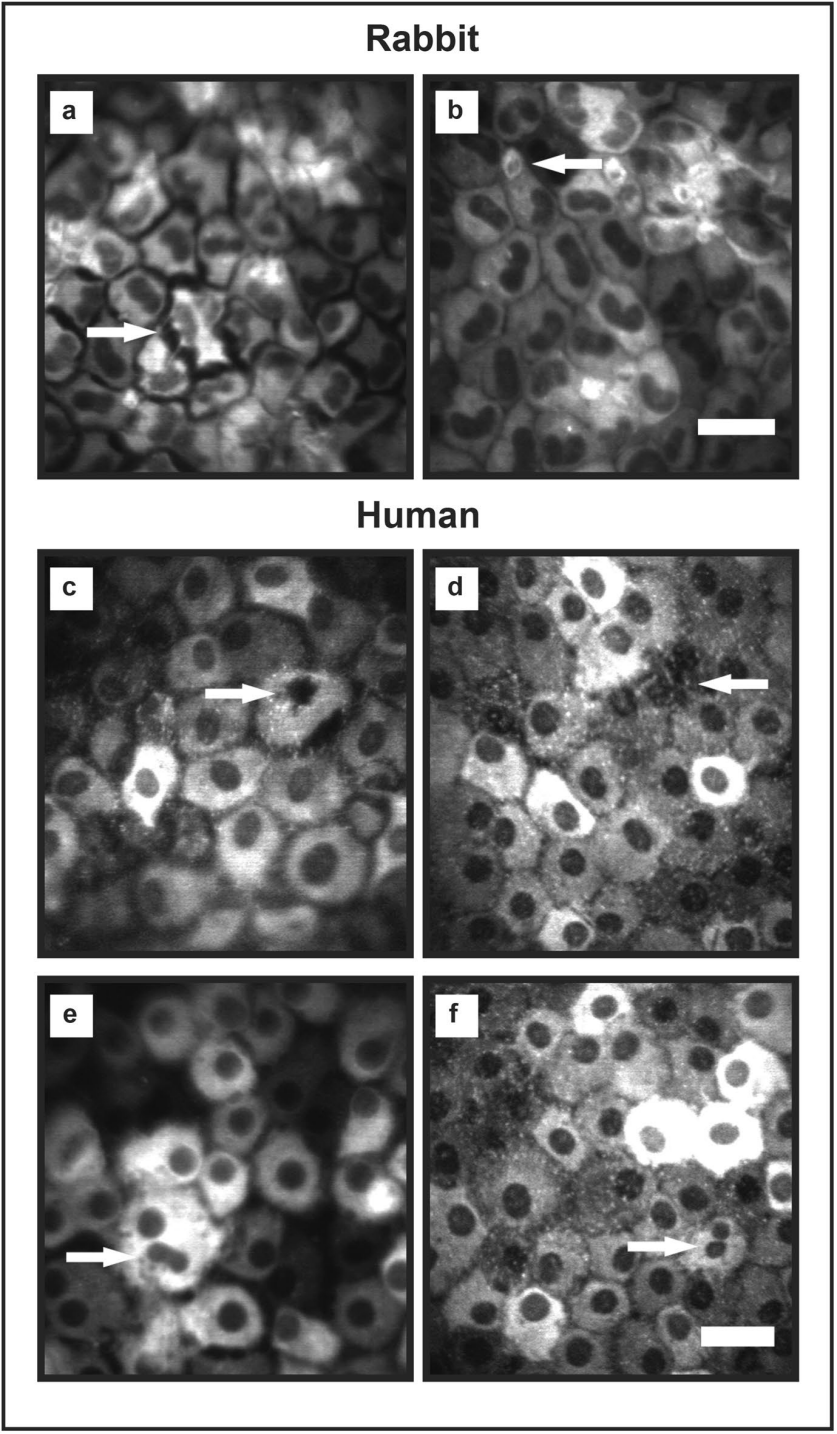
High-resolution images achieved with Spectralis and Rostock module. High-resolution images of rabbit endothelial cells (stained with DIR by intracameral injection) and human endothelial cells (from EK transplants stained with DIR) are achievable with the HRA/Spectralis and Rostock module. (**a**) A rabbit endothelial cell can be seen detaching from the monolayer. (**b**) Small, possibly inflammatory, cells staining positive for DIR are visible interspersed with the rabbit endothelial cells. (**c**) A human endothelial cell undergoing apoptosis exhibits a pyknotic nucleus and separation from adjacent cells. (**d**) Podocyte extension to fill a gap in the monolayer following cell loss can be seen. (**e**) Bi-lobulated nuclei and (**f**) smaller adjacent nuclei suggestive of paired daughter cells are suggestive of in vivo cell division. Scale bar, 50 µm.

By attaching the Rostock module (Heidelberg Engineering, Heidelberg, Germany) to the HRA/Spectralis, we were able to acquire detailed, high-resolution in vivo images. This allowed us to observe subtle biological processes at a cellular level, including nuclear fragmentation and cell detachment in both rabbit (Fig. 11a,b) and human (Fig. 11c) corneal endothelium; podocyte extension from neighboring cells (Fig. 11d); nuclei in anaphase (Fig. 11e) and daughter cell formation (Fig. 11f).

## Discussion

Tracking of CECs in vivo is not widely performed to date due to a lack of suitable imaging techniques and paucity of data on labelling efficiency, duration of fluorescence, general toxicity, and effects of labelling on functionality of transplanted tissue^21^. The aim of this study was to determine whether NIR dye, and specifically for this study, DIR, could be used to label and track CECs, in vivo, without impacting cell viability or cellular function. We began by assessing the effect of DIR staining on wound healing, cell migration and cell viability, in vitro, using cultured primary human CEC’s. At the concentrations required for in vivo imaging, DIR stained CECs migrated at the same rate as unlabeled CECs. At 24 h post-labelling, there was no significant difference in cell death between labelled and unlabeled cells. Taken together, these results suggest that DIR was a viable candidate for use as a tracker in CECs. We subsequently assessed whether use of DIR had any deleterious effects in vivo. A single intracameral DIR injection was sufficient to label rabbit endothelial cells and was not associated with any apparent inflammation or acute disturbance of corneal endothelial function. We subsequently used DIR imaging to track the endothelial cells on EK transplants from human tissue. We found it was possible to obtain high quality images of human endothelial cells within the anterior chamber following EK. To our knowledge, this is the first time biological processes such as healing of iatrogenic wounds and migration of endothelial cells from donor to host have been observed in vivo.

At the macroscopic level, we found DIR imaging to be comparable to repeated trypan blue injection when assessing endothelial wound healing following standard injury; a model commonly used to assess pharmacological therapies aimed at enhancing endothelial regeneration^22–24^. High magnification assessment of the healing edge of the wound showed that comparable morphological information could be obtained from in vivo imaging as compared to immuno-histology following animal sacrifice. A temporal relationship between closure of endothelial wounds, as observed by DIR imaging, and restoration of corneal clarity/CCT was also seen in all cases. In vivo wound healing kinetics were similar to those we previously reported in unlabeled cells^10^. Images could be acquired through opaque/edematous corneas, something not possible with conventional confocal imaging. Combined, these findings suggest that DIR staining has no significant effect on endothelial function or healing and allows continuous, in vivo, assessment at all time points post-wounding. Assessment of endothelial transplants stained with DIR showed no diffusion of DIR across cell membranes from adjacent endothelial cells, meaning that staining of rabbit endothelial cells did not occur following xenotransplantation with human DMEK grafts (Fig. 8). This allowed for a clear distinction between transplanted human endothelial cells and native rabbit endothelial cells, even if cells had migrated on or off the transplant itself.

We have previously shown that rabbit endothelial cells are capable of rapidly migrating over bare DM with subsequent restoration of corneal clarity and thickness to pre-transplantation levels^10^. As such, it is important to be certain that any restoration of function is attributable to the transplanted cells and not a consequence of cellular regeneration in models where in vivo endothelial replication occurs^25^. Until now, detailed assessment of wound healing at different time points has relied either on repeated surgical interventions, in the case of trypan blue injection^10^, or on the sacrifice of animals at multiple time points^26^. Our assessments showed that a DIR fluorescence signal, allowing for high quality image acquisition, was retained for at least 28 days and in subsequent experimentation we have found fluorescence to be retained up to 60 days (data not shown).

With high resolution imaging, biological processes typically observed on histological examination could be seen in vivo; including cell spreading, apoptosis and cell division. In one animal, a reduction in corneal clarity, together with a development of corneal edema and an increase in corneal thickness occurred between days 21 and 28. DIR imaging showed numerous endothelial cells had detached from the DM and were freely floating in the aqueous. The cells remaining attached to DM displayed features consistent with an acute insult^13^. In the setting of xenotransplantation, these features were consistent with acute graft rejection^27,28^. In keeping with this, small DIR positive cells, consistent with previous reports of inflammatory cells, were observed interspersed with the endothelial cells were seen in both in the rabbit endothelial monolayer and transplanted human endothelial cells^29^.

Graft rejection remains a leading cause of transplant failure. Currently, models of rejection rely on indirect measures of endothelial function, and it is not always possible to separate rejection from other forms of endothelial trauma^11,30^. Methods of imaging inflammatory cell changes that proceed acute rejection in vivo will allow a greater understanding of the mechanisms at play^31^. Being able to assess the degree of cell death occurring during a rejection episode will provide information on the likelihood of recovery following rejection and effectiveness of anti-rejection strategies.

The use of NIR markers for tracking has several advantages. These tracers benefit from very low autofluorescence at NIR wavelengths and have excellent tissue penetration. This combination gives a high signal-to-noise ratio and allows non-invasive tracking of cells through the skin in organs such as the spleen or heart^32^. NIR tracers have recently been used in surgical trials to aid tumor clearance^33^ and in clinical ophthalmic trials to assess cell loss in patients with early glaucoma^34^. Using an 800 nm band pass, sufficient emitted light is captured for high quality imaging, and this is possible even through opaque corneas with significant edema. The NIR dye, indocyanine green (ICG), has been used in ophthalmology for over 5 decades. This means that dedicated equipment for imaging of NIR tracers in the eye are widely available in clinical and research settings. Our imaging protocol uses clinical equipment, designed for ICG imaging, with minimal adjustments.

Given that DIR staining is a simple, non-toxic, one step process, it is possible that this method could be applied to graft tissue destined for human use if a good manufacturing practice form of the dye were made available. For organ cultured tissue, the point at which graft tissue is transferred to dextran containing media may provide an ideal opportunity for this^35^. It is possible that DIR may also be useful to track other cells used in corneal regenerative therapy such as limbal epithelial^21^ or stromal stem cells^36^.

Our study showed that DIR fluorescence was initially quite heterogenous. This meant multiplexing with the viability dye, Calcein AM, was needed to assess immediate post-op graft viability. Fluorescence peaked at 24 h post transplantation making imaging easier, although some heterogeneity remained, meaning appropriately exposing the images was challenging. It may be possible to improve the use of DIR by combining several images taken at different sensitivities (iso settings); high dynamic range imaging^37^.

We believe that DIR imaging provides a powerful new tool for studying CECs in vivo. More detailed information about endothelial cell behavior following transplantation will improve our understanding of phenomena such as accelerated cell loss in high risk grafts and may lead to targeted therapeutics and improved graft survival. Its use in a research setting should ultimately lead to a reduction in the number of research animals needed and there is a potential for easy adoption in the clinical setting. Further studies confirming the exact length of time that DIR fluorescence persists in-vivo are warranted.

## Methods

### Study tissue

The use of cadaveric donor corneas for this study was approved by Singhealth centralized institutional review board (Ref: 2016/2839), and all cell-based research performed with human derived tissue was carried out in accordance with the tenets and the principles outlined in the Declaration of Helsinki. For the expansion of primary CECs, research grade donor corneas with endothelial cell counts of > 1800 cells/mm^2^ with a storage time of < 14 days in Optisol-GS (Bausch & Lomb Rochester, New York) were procured from either Lions Eye Institute for Transplant and Research (Tampa, Florida, USA) or Saving Sights (Kansas City, Missouri, USA), with informed consent from the next of kin. The isolation and subsequent propagation of the primary CECs was achieved using a dual media approach as described^38,39^. Briefly the DM is peeled from the donor cornea, and subsequently subjected to a two-step enzymatic treatment to release the CECs from the DM. Isolated cells were seeded onto pre-coated collagen culture plate at a seeding density of at least 1.0 × 10^4^ cells/cm^2^ and established in a maintenance medium (M5-Endo; Human Endothelial-SFM supplemented with 5% serum) overnight. Subsequently, CECs were cultured in the proliferative medium (M4-F99; Ham’s F12/M199, 5% serum, 20 μg/ml ascorbic acid, 1× ITS, and 10 ng/ml HrFGF) until they are approximately 80% confluent, before being re-exposed to M5-Endo for at least two days. The expanded CECs were then sub-cultured via single-cell dissociation using TrypLE Select, and plated at the desired seeding density for optimization studies. All cultured were carried out in a humidified atmosphere at 37 °C and 5% CO_2_.

For transplantation study, human corneo-scleral buttons with consent for research use were obtained from Miracles in Sight (Winston Salem, North Carolina, USA). Tissue preparation and transplantation was performed by a single surgeon (MB). Transplant grade tissue with endothelial cell counts of > 2200 cells/mm^2^ and a storage time < 14 days in Optisol-GS was used for all experimentation.

### Research animals

Animals procured for this study were treated in accordance with the ARVO Statement for Use of Animal in Ophthalmic and Visual Research as well as the ARRIVE guidelines^40^, and all experiments involving these animals were approved by the Singhealth Institutional Animal Care and Use Committee (IACUC; 2016/SHS/1212), Singapore. Interventions were performed in New Zealand white rabbits aged 16–20 weeks, weighing between 3 and 3.5 kg. All surgical procedures and clinical evaluations were performed in a single eye of each rabbit under general anesthesia from intra-muscular xylazine 5 mg/kg (Troy Laboratories, Smithfield, Australia) and ketamine 40 mg/kg (Parnell Laboratories, Alexandria, Australia) with additional topical anesthesia (lidocaine 1%, Bausch and Lomb, Rochester) as previously described^10^.

Animals underwent phacoemulsification at least 1 week prior to endothelial keratoplasty to deepen the anterior chamber. Vancomycin was used in the irrigating fluid (20 µg/ml) for surgical procedures and rabbits received subconjunctival dexamethasone (1 mg) and gentamcin (0.4 mg) at the end of all surgical procedures.

Prior to EK, rabbits were injected with intravenous unfractionated heparin (1000 units in 1 ml of normal saline). Heparin was also added to the balanced salt solution used to irrigate the eye at a concentration of 50 units/ml. For all experiments, rabbits were examined daily for the first 5 days and then on days 7, 14, 21, and 28. Examinations consisted of slit lamp biomicroscopy, tonometry, optical coherence tomography, confocal fluorescence imaging using the Heidelbreg HRA/Spectralis (Heidelberg Engineering GmbH, Heidelberg, Germany) and conventional corneal confocal imaging using the Heidelberg HRT-II with the Rostock module (Heidelberg Engineering GmbH, Heidelberg, Germany).

### Optimizing DIR concentration in vivo

A stock solution of 1,1′-dioctadecyl-3,3,3′,3′-tetramethylindotricarbocyanine iodide (DIR) dye was made by dissolving 10 mg of DIR powder in 1 ml of pure ethanol (ThermoFisher Scientific, USA).

Primary cultured CECs were seeded at a concentration of 2 × 10^5^ cells/cm^2^ in a Greiner 48-well flat bottom polystyrol microplate (Greiner, Frickenhausen, Germany), pre-coated with FNC coating mix. Cells were incubated with DIR diluted in M5 media^39^ at a concentration of 10–200 µg/ml overnight at 37 °C (N = 15 per concentration). The following day, cells were washed thrice in fresh culture media and imaged using the HRA/Spectralis at a standardized sensitivity of 100. Images were exported to ImageJ (National Institute of Health, Bethesda, USA) and converted to 8-bit. Mean grey values were measured in three separate regions of each plate and average fluorescence for each dose calculated. A comparison between groups was made using ANOVA.

### Toxicity of DIR in vitro

DIR-labelled endothelial cells and controls were cultured for a further 48 h in fresh media. Calcein AM/ethidium homodimer/Hoechst were added to the cultured cells to achieve final concentrations of 2 µM, 4 µM and 10 µM respectively. The plate was gently agitated, incubated at 37 °C for 30 min and imaged on the fluorescence microscope. The viable ECD was calculated and compared between control and treated cells. A positive control was performed by using the same viability assessment in cells incubated with 70% ethanol for 1 min prior to assessment. A comparison between groups was made using ANOVA.

### Effect of DIR on endothelial cell migration

As the primary aim of DIR tracking was to examine cell migration, a scratch wound assay was performed. Primary cultured CEC’s from the same donors were seeded onto different wells within the same plate. After reaching confluence, cells were cultured in M5 culture media with DIR 100 µg/ml or M5 media alone (controls). Each well received a single scratch using the tip of a 10 µl pipette. Plates were placed within the incuCyte imaging incubator (Sartorius, Essen Bioscience, Ann Arbor, Michigan, USA). The incuCyte is a multiplex imaging system that is incorporated into an incubator, allowing continuous imaging in multiple wells without alterations in temperature or atmosphere. Image registration to define the scratch area was performed and serial phase-contrast images were taken every hour for 24 h. The wound area was measured every 3 h and expressed as a percentage of that measured at T = 0 h. Wound size was compared between stained cells and unstained controls using multiple t-tests.

### Labelling of rabbit endothelial cells in vivo

0.2 ml of DIR, diluted in BSS at a concentration of 200 µg/ml was drawn into an insulin syringe and the needle inserted into the anterior chamber at the limbus, taking care to tunnel the entry for at least 1 mm. A 1.0 mm paracentesis was made using a diamond knife. 0.2 ml of aqueous was drawn into the insulin syringe achieving a final concentration of 100 µg/ml DIR. The posterior lip of the paracentesis was depressed causing expression of the aqueous all of the aqueous, after which the DIR was slowly injected thus performing a complete DIR/aqueous exchange and ensuring a concentration of not greater than 100 µg/ml. It is possible the final concentration was a little lower due to the inability to remove all of the aqueous and its rapid replenishment. The rabbits were kept with the treated eye facing down until the anaesthesia had worn off (approximately 45 min).

### Imaging of endothelial cells post-operatively

Imaging was performed using the HRA/Spectralis set in the ICG mode. In the ICG mode, the HRA/Spectralis utilizes a 790 nm diode excitation laser and 800 nm long-pass filter. Images were captured at varying levels of magnification by attaching microscope objective lenses (Nikon, Japan) or the Rostock module to the standard fundus imaging lens by using custom built collars, as previously described^3^. Use of multiple lenses allowed both macroscopic and individual cell level imaging.

### Comparison of DIR imaging and established methods in a wound healing model

Two weeks following DIR injection, 9 rabbits had 8 mm endothelial scrape wounds as described in our previous work^10^. 6 rabbits were sacrificed at day 3, one at day 5 and the remaining two animals were sacrificed at day 28. Pan-corneal images were acquired using the manufacture supplied anterior segment lens for the HRA/spectralis. These allowed tracking of the entire wound areas, macroscopically.

For higher magnification imaging, the inferior edge of the wound was located by moving the lens array up from the inferior limbus until the leading edge of migrating cells was seen, allowing a consistent area to be sampled for DIR and conventional in vivo confocal microscopy. In rabbits sacrificed at day 3, the endothelial wound was assessed by injecting trypan blue into the anterior chamber as previously described^5^. Trypan blue and DIR images were exported to ImageJ and the wound outlined using the polygon selection tool. This was converted to a smoothed spline and measurements of wound area and circularity were taken. The measurements for imaging technique were compared using a Bland–Altman plots.

After killing, the inferior cornea was marked by passage of a 6-0 silk suture through the limbus at the 6 o’clock position and used as a reference for immunohistology.

The corneas were harvested, washed in BSS and fixed in 1% paraformaldehyde at room temperature for 5 min. Samples were permeabilized in Triton-X 1% for 5 min and then blocked using and 2 step process, first in 10% goat serum for 45 min followed by 3% BSA for 45 min. Samples were incubated with primary antibody to ZO-1 (1:300, Thermofischer) or Human Nuclear Antigen (1:100, Merck-Millipore) at 4 °C for 24 h in a solution composed of phosphate buffered saline (PBS) with 0.1% Triton-X and 1% BSA. Samples were washed 3× with PBS, each for 10 min, and then incubated with secondary antibody, Hoechst (1:1000) and phalloidin (1:500), as required, overnight at 4 °C. To assess specificity of the immunostaining, corneas were processed without primary antibody. Specimens were washed 3× in PBS, re-fixed in 3% PFA, washed again and flat mounted in ProLong gold. The entire cornea was flat mounted and the inferior edge of the wounded area, in line with the silk suture was identified for imaging. Individual cells were manually outlined and cell metrics compared between in vivo images and flat-mounted, immunofluorescence images (stained with phalloidin, Hoechst and ZO-1). At day 3, when cells were still migrating, a comparison between cell circularity was made (a minimum of 100 cells were measured in each group). At day 28 when a stable monolayer was present, a comparison between cell circularity and the percentage of hexagon cells was made (neighbor analysis function in the BioVoxxel toolbox for ImageJ).

### Labelling of ex vivo human corneas

Human corneas were labelled prior to tissue preparation for Descemet Membrane Endothelial Keratoplasty (DMEK) and following mircokeratome cutting for Descemet Stripping Automated Endothelial Keratoplasty (DSAEK). The procedure was optimized in a series of initial experiments. To limit the amount of dye required and prevent corneal swelling, the corneas were kept within the Optisol-filled viewing chamber. Optisol was aspirated from the corneal cup and the endothelial surface further dried with cellulose sponges. DIR (100 µg/ml), diluted in M5 medium, was pipetted onto the endothelial surface until the entire endothelium and trabecular meshwork were covered. The epithelial surface remained suspended by the pillars of the viewing chamber and in contact with the Optisol, with the scleral rim acting as barrier to prevent the two media for mixing. The lid for the viewing chamber was replaced loosely and the corneas were incubated over night at 37 °C. For corneas in which a comparison between DIR and Calcein AM fluorescence was performed, corneas were rinsed 3× in fresh M5 media and the incubated with Calcein AM 1.67 µM in BSS for 30 min prior to surgery.

Tissue was prepared and implanted using our previously described techniques for DMEK (n = 4)^5,41^ and DSAEK (n = 6)^42^ and imaging for Calcein AM and DIR fluorescence performed alongside the standard examinations, following the review schedule outlined above.

### Statistical analysis

All statistical analysis was conducted using Prism (Graphpad software LLC, San Diego, USA). Unless otherwise stated significance was set at 5%. For multiple comparisons a Bonferroni adjustment was performed.

## Supplementary Information

Supplementary Video S1.

## Abbreviations

CECs: Corneal endothelial cells
ECD: Endothelial cell density
CCT: Central corneal thickness
OCT: Optical coherence tomography
EK: Endothelial keratoplasty
NIR: Near-infrared
DIR: 1,1′-Dioctadecyl-3,3,3′,3′-tetramethylindotricarbocyanine iodide
DM: Descemet’s membrane
DMEK: Descemet membrane endothelial keratoplasty
DSAEK: Descemet stripping automated endothelial keratoplasty
ICG: Indocyanine green
